# Painful faces-induced attentional blink modulated by top–down and bottom–up mechanisms

**DOI:** 10.3389/fpsyg.2015.00695

**Published:** 2015-06-01

**Authors:** Chun Zheng, Jin-Yan Wang, Fei Luo

**Affiliations:** ^1^Key Laboratory of Mental Health, Institute of Psychology, Chinese Academy of Sciences, BeijingChina; ^2^University of Chinese Academy of Sciences, BeijingChina; ^3^Department of Psychology, Southwest University for Nationalities, ChengduChina

**Keywords:** attentional blink, bottom–up, pain, RSVP, top–down

## Abstract

Pain-related stimuli can capture attention in an automatic (bottom–up) or intentional (top–down) fashion. Previous studies have examined attentional capture by pain-related information using spatial attention paradigms that involve mainly a bottom–up mechanism. In the current study, we investigated the pain information-induced attentional blink (AB) using a rapid serial visual presentation (RSVP) task, and compared the effects of task-irrelevant and task-relevant pain distractors. Relationships between accuracy of target identification and individual traits (i.e., empathy and catastrophizing thinking about pain) were also examined. The results demonstrated that task-relevant painful faces had a significant pain information-induced AB effect, whereas task-irrelevant faces showed a near-significant trend of this effect, supporting the notion that pain-related stimuli can influence the temporal dynamics of attention. Furthermore, we found a significant negative correlation between response accuracy and pain catastrophizing score in task-relevant trials. These findings suggest that active scanning of environmental information related to pain produces greater deficits in cognition than does unintentional attention toward pain, which may represent the different ways in which healthy individuals and patients with chronic pain process pain-relevant information. These results may provide insight into the understanding of maladaptive attentional processing in patients with chronic pain.

## Introduction

As an alarm signal of bodily threat, pain has an inherently stronger ability than other stimuli to draw attention and interrupt ongoing goals (Eccleston and Crombez, 1999; Legrain et al., 2009; Van Damme et al., 2010; Schoth et al., 2012). Similarly, pain-related information also has the advantage of capturing attention preferentially, consuming attentional resources, and making attention switching difficult. Using the dot-probe task (Roelofs et al., 2005; Liossi et al., 2009; Haggman et al., 2010; Baum et al., 2013) and spatial cueing paradigm (Van Damme et al., 2004a,b, 2006b), researchers have found significant attentional bias toward painful words/faces and visual cues predictive of pain compared with neutral words/faces and non-pain cues, respectively. These studies have examined attentional bias toward pain-related stimuli based on the spatial distribution of attention. Few studies have addressed these issues in terms of the temporal dynamics of attention. Moreover, available evidence supporting pain-related attentional bias comes largely from patients with chronic pain; this bias is rarely found in healthy subjects (Crombez et al., 2013).

The AB paradigm is used widely to study the temporal deployment of attention (Martens and Wyble, 2010). In a typical AB paradigm, two targets are embedded in a stream of items in a RSVP. The AB effect is characterized by a failure to detect the second target, which follows the first target after a very short interval (Raymond et al., 1992; Maciokas and Crognale, 2003). This paradigm has been used to explore the effect of emotional information on attention capture (de Oca et al., 2012; Schonenberg and Abdelrahman, 2013; Yerys et al., 2013; de Jong et al., 2014). Recent studies using a single-target RSVP task have found that task-irrelevant emotional words (Barnard et al., 2005; Arnell et al., 2007) or pictures (Kennedy and Most, 2012; Quaedflieg et al., 2013) are capable of inducing an AB. Most et al. (2005) defined the difference in task performance when viewing emotional and neutral pictures as emotion-induced AB (Smith et al., 2006).

Pain-related information can capture attention in a bottom–up or top–down fashion (Crombez et al., 2005; Van Damme et al., 2010). Most previous studies investigating pain-related attention capture have used task-irrelevant stimuli (i.e., stimulus-driven or bottom–up mode; Van Damme et al., 2010; Schoth et al., 2012; Sharpe, 2012; Crombez et al., 2013). No study other than that conducted by Grynberg et al. (2014) has employed pain-related material as a task-relevant stimulus (i.e., goal-driven or top–down mode). Distinct patterns of attentional processing of pain-related stimuli have been thought to represent different ways in which healthy individuals and patients with chronic pain process pain-relevant information, with the former using mainly a stimulus-driven, bottom–up pattern and the latter using mainly a top–down process (Van Damme et al., 2010; Crombez et al., 2013). Selective attention to and difficulty in disengaging from pain-related information lead to cognitive impairment, as well as the maintenance or exacerbation of pain, in patients with chronic pain (Vlaeyen and Linton, 2000; Leeuw et al., 2007). No study to date has compared the effects of bottom–up and top–down attention engagement using pain-related information. Thus, the present study aimed to examine the effects of these two attention modes using task-irrelevant and task-relevant AB paradigms, to provide a possible basis for the understanding of maladaptive attentional processing in patients with chronic pain.

This study involved the administration of a single-task RSVP, in which only a target image was identified, and a dual-task RSVP, in which subjects were asked to respond to a distractor and a target, to healthy subjects. Photographs depicting painful and neutral facial expressions were used as distractors. Subjects were asked to complete the self-reported PCS and IRI. Pain catastrophizing is believed to play an important role in shaping the experiences of acute and chronic pain, and has been shown to be associated with attentional bias for pain-relevant stimuli (Van Damme et al., 2002, 2004a). The IRI measures trait empathy; human elements such as faces and voices have been suggested to provide important cues triggering empathy (Jabbi et al., 2007; Choi and Watanuki, 2014). The following two hypotheses were tested: (1) pain information-induced AB effects (i.e., worse target performance in the painful condition than in the neutral condition) would be observed for both tasks, and would be more significant in the top–down (dual task) model than in the bottom–up (single task) model; and (2) pain catastrophizing and/or empathy trait would be related to the target accuracy in pain condition.

## Materials and Methods

### Participants

Fifty-two healthy, right-handed undergraduate and graduate students, with normal or corrected-to-normal vision, participated in this study (mean age 22.0 ± 2.6 years, 35 females). All participants had no history of psychiatric or neurological disorders, and none of them reported current pain or a past history of chronic pain. The Institutional Review Board of the Institute of Psychology, Chinese Academy of Sciences, approved the experimental procedures. Informed consent was obtained from each subject before the experiment. All individuals were paid for participation.

### Experimental Procedure

**FIGURE 1:**
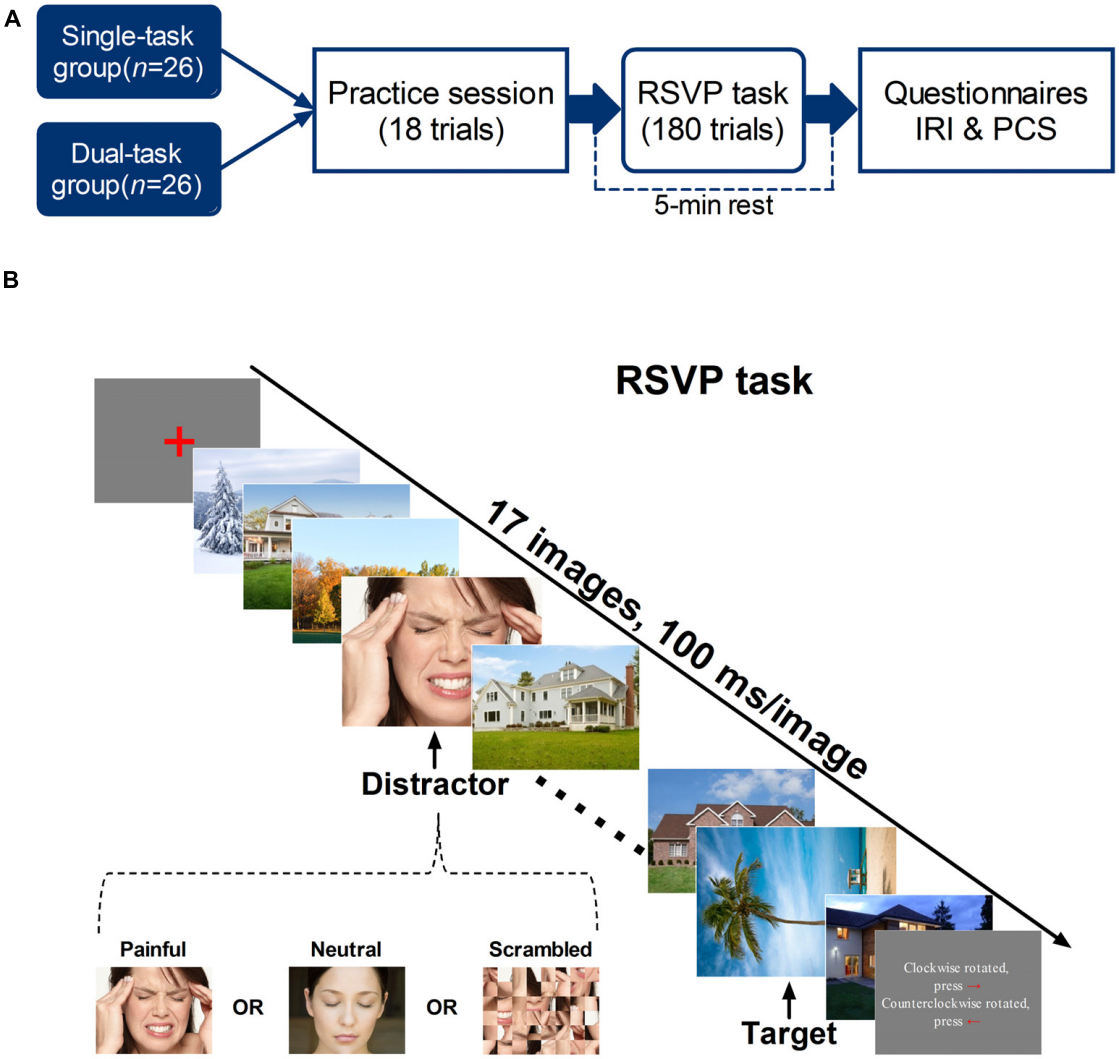
**(A)** Experimental procedure. Participants were randomly assigned to a single-task RSVP group, in which only one target was identified, or a dual-task RSVP group, in which participants were instructed to respond to a distractor and a target. After finishing 18 practice trials, the experimental session began, in which participants performed 180 RSVP trials. At the end of the tasks, participants completed the IRI and PCS. **(B)** Example of an RSVP trial. Each trial began with the presentation of a red fixation cross for 100–300 ms (random duration) in the center of the gray background. The subsequent RSVP involved the presentation of 17 images for 100 ms each. Depending on the trial, the fourth, sixth, or eighth stimulus was the distractor: a painful, neutral, or scrambled photograph. Each trial also included a target: a tree/architectural photograph rotated 90° clockwise or counterclockwise, which appeared either two or eight items (lag 2 and lag 8, respectively) after the distractor. In the single task, participants were instructed to indicate the target’s orientation and ignore the distractor; in the dual task, participants were required to respond to both.

Experiments were performed in a sound-attenuated room with the temperature maintained at 26°C, relative humidity of 48–53%, and luminance level of 361–374 lux. Participants were seated in a comfortable chair at a viewing distance of 60 ± 5 cm from a computer monitor. An RSVP paradigm was delivered to measure the attention-capturing effect of pain-related information. Participants were randomly assigned to a single-task RSVP group (*n* = 26, 18 females), in which only one target was identified, or a dual-task RSVP group (*n* = 26, 17 females), in which participants were instructed to respond to a distractor and a target. Each subject was allowed to practice before the formal experiment. After terminating the task, participants were asked to complete the IRI and PCS. **Figure 1A** illustrates the experimental procedure.

### Visual Stimuli

The stimuli were presented in the center of a gray background on a 17-inch cathode ray tube monitor with a refresh rate of 100 Hz using E-prime 1.1 software (Psychology Software Tools, Inc., Pittsburgh, PA, USA). The screen resolution was set to 800 × 600 pixels, resulting in a photograph display size of about 13.8 cm × 10.3 cm.

Visual stimuli were 320 × 240-pixel color photographs gathered from the Internet (http://www.gettyimages.cn). The stimuli consisted of 180 photographs of adult male and female faces (distractors; 60 painful, 60 neutral, and 60 scrambled), 180 photographs of tree/architectural elements (targets; 90 rotated 90° clockwise and 90 rotated 90° counterclockwise), and 240 upright tree/architectural images (fillers). The painful distractors depicted men and women with painful expressions and hands on the forehead, indicating headache. The painful and neutral faces were matched by gender. The scrambled distractors were created by segmenting each painful image using an 8 × 6 grid and randomly reordering the segments using Adobe Photoshop CS4 (Adobe Systems, Inc., San Jose, CA, USA). The scrambled pictures served as controls to ensure that the behavioral differences elicited by painful and neutral conditions were not due to low-level visual properties, such as color and luminance. **Figure 1B** depicts an example of an RSVP image stream. Each target and distractor image appeared only once during the task.

Prior to the experiment, subjects not involved in the main study rated the degrees of pain and arousal depicted in facial photographs on a seven-point scale ranging from 0 (“no feeling” and “calm” respectively) to 6 (“intolerably painful” and “excited” respectively). An initial sample of 240 face photographs was divided into two sets, each containing 120 photographs and rated by an independent group of subjects (*n* = 20, 10 females) to prevent fatigue. The ages of subjects in the two groups were matched (22.1 ± 3.3 and 22.6 ± 1.4 years; *t*_(38)_ = 0.57, *p* = 0.57). A total of 120 face photographs with the highest and lowest pain scores (*n* = 60 each) were used as painful and neutral distractors, respectively, in the present study. Subjective ratings of these painful and neutral distractors were shown to differ significantly before the formal experiment was conducted (pain score: 3.67 ± 0.53 vs. 0.47 ± 0.12, *t*_(118)_ = 45.52, *p* < 0.001; arousal score: 3.20 ± 0.32 vs. 2.47 ± 0.36, *t*_(118)_ = 11.68, *p* < 0.001).

### Rapid Serial Visual Presentation Paradigm

The experiment comprised 180 RSVP trials. Participants started trials by pressing the space bar on the computer keyboard. Each trial began with the presentation of a fixation cross (random duration, 100–300 ms) in the center of the monitor. The RSVP trial consisted of a stream of 17 images, including a distractor image, a target image, and 15 filler images, each presented for 100 ms. Distractors were positioned as the fourth, sixth, or eighth image in each trial, similar to the method used by Most et al. (2005). Distractor position was randomized across trials. The target appeared two (lag 2, SOA = 200 ms) or eight (lag 8, SOA = 800 ms) places after the distractor. **Figure 1B** illustrates the sequence of events for one RSVP trial. Participants were required to provide responses after presentation of the stream, at their own pace. One second after responding, participants could press the space bar again to start the next trial.

The single-task RSVP was preceded by a brief practice session (18 trials) that included no distractor. Targets were similar to those used in the experiment, and filler images were selected from the set used in the experiment. Each item in the RSVP stream was presented for 200 ms to ensure that participants could identify the target and learn the rules of the experiment. In the dual-task RSVP, participants followed the same procedure as in the single-task experiment, but they were given different instructions (see Instructions). The initial 18-trial practice session was similar to that preceding the single-task RSVP, but it contained distractor images (similar to those used in the experiment). No feedback was provided to participants.

### Instructions

For the single-task RSVP, participants were given the following instructions: “You will see a series of rapidly presented photographs of landscapes and faces. You should pay attention to a rotated photo and indicate its orientation by pressing the corresponding (left or right) arrow key. Note that only one landscape photo is rotated.”

Instructions for the dual-task RSVP were: “You will see a series of rapidly presented photographs of landscapes and faces. You should pay attention to both a face photo and a rotated landscape photo, and identify the type of face by pressing the corresponding key on the keyboard (“1” = painful, “2” = neutral, “0” = scrambled). Then, indicate the orientation of rotated photo by pressing the corresponding (left or right) arrow key. Note that only one landscape photo is rotated.”

### Interpersonal Reactivity Index

The IRI was developed by Davis (1980). It comprises 28 items rated on a five-point scale, and measures trait empathy using four subscales. The perspective-taking subscale assesses the tendency to spontaneously adopt others’ psychological points of view; the fantasy subscale measures respondents’ tendency to transpose themselves imaginatively into the feelings and actions of fictitious characters in books, movies, and plays; the empathic concern subscale assesses “other-oriented” feelings of sympathy and concern for unfortunate others; and the personal distress subscale measures “self-oriented” feelings of personal anxiety and unease in tense interpersonal settings. The IRI total score is the sum of all subscale scores. The IRI subscales have been shown to have good internal consistency (ranging between 0.71 and 0.77; Davis, 1980, 1983). The Chinese version of the IRI, used in the present study, has been demonstrated to have satisfactory construct reliability (0.59, 0.75, 0.60, and 0.69 for perspective-taking, fantasy, empathic concern, and personal distress subscales, respectively) and retest reliability (0.64, 0.75, 0.59, and 0.78 for perspective-taking, fantasy, empathic concern, and personal distress subscales, respectively; Rong et al., 2010).

### Pain Catastrophizing Scale

The PCS was designed to measure catastrophic thinking about pain (Sullivan et al., 1995). It contains 13 items in three subscales: rumination (e.g., “I can’t stop thinking about how much it hurts”), magnification (e.g., “I worry that something serious may happen”), and helplessness (e.g., “It’s awful and I feel that it overwhelms me”). Each item is rated on a five-point scale. The PCS total score is the sum of all subscale scores. The PCS has been shown to have good internal consistency (Cronbach’s α = 0.87 for the total PCS, 0.87 for rumination, 0.60 for magnification, 0.79 for helplessness; Sullivan et al., 1995). The Chinese version of the PCS (HK-PCS), used in the present study, has been demonstrated to have satisfactory internal consistency (Cronbach’s alpha = 0.927, 0.809, 0.768, and 0.839 for the total scale and rumination, magnification, and helplessness subscales, respectively; Yap et al., 2008).

### Data Analyses

For both tasks, the accuracy of target direction judgment was recorded for analysis. For the dual-task group, only trials in which distractors were identified correctly were included in the analysis. The pain information-induced AB was defined as the difference in target accuracy between the neutral and painful conditions at lag 2.

All statistical analyses were performed using SPSS 13.0 (SPSS, Inc., Chicago, IL, USA). The accuracy of face identification in the dual task was assessed using 3 × 2 (distractor type × lag) repeated-measures ANOVA. Task performance (i.e., target accuracy) data were entered into a 2 × 3 × 2 [task (single, dual) × distractor type (painful, neutral, scrambled) × lag (lag 2, lag 8)] mixed-design ANOVA, with repeated measures on the last two factors. Split-up ANOVAs were performed in cases of three-way and two-way interaction. Separate 3 × 2 (distractor type × task) ANOVAs were conducted for lag 2 and lag 8 data. When the ANOVA of lag 2 data showed significant interaction, one-way ANOVA was conducted for distractor type. *Post hoc* comparisons were performed using Fisher’s least significant difference method. Effect sizes for ANOVAs were reported using partial eta squared (η^2^) values. Pearson’s correlations were computed to explore relationships between target accuracy and IRI and PCS scores. The significance level was set at *p* < 0.05.

## Results

### Performance of Face Identification in the Dual Task

For the dual task, the accuracy of identification of painful, neutral, and scrambled faces was 93 ± 7, 95 ± 6, and 95 ± 4%, respectively, at lag 2 and 95 ± 8, 96 ± 6, and 97 ± 4%, respectively at lag 8. Two-way ANOVA revealed no significant main effect (lag: *F*_(1,25)_ = 1.57, *p* = 0.22, η^2^ = 0.06; distractor type: *F*_(2,50)_ = 1.26, *p* = 0.29, η^2^ = 0.05) or interaction (distractor type × lag: *F*_(2,50)_ = 0.17, *p* = 0.79, η^2^ = 0.01).

### Pain Information-Induced Attentional Blink Effect

**FIGURE 2:**
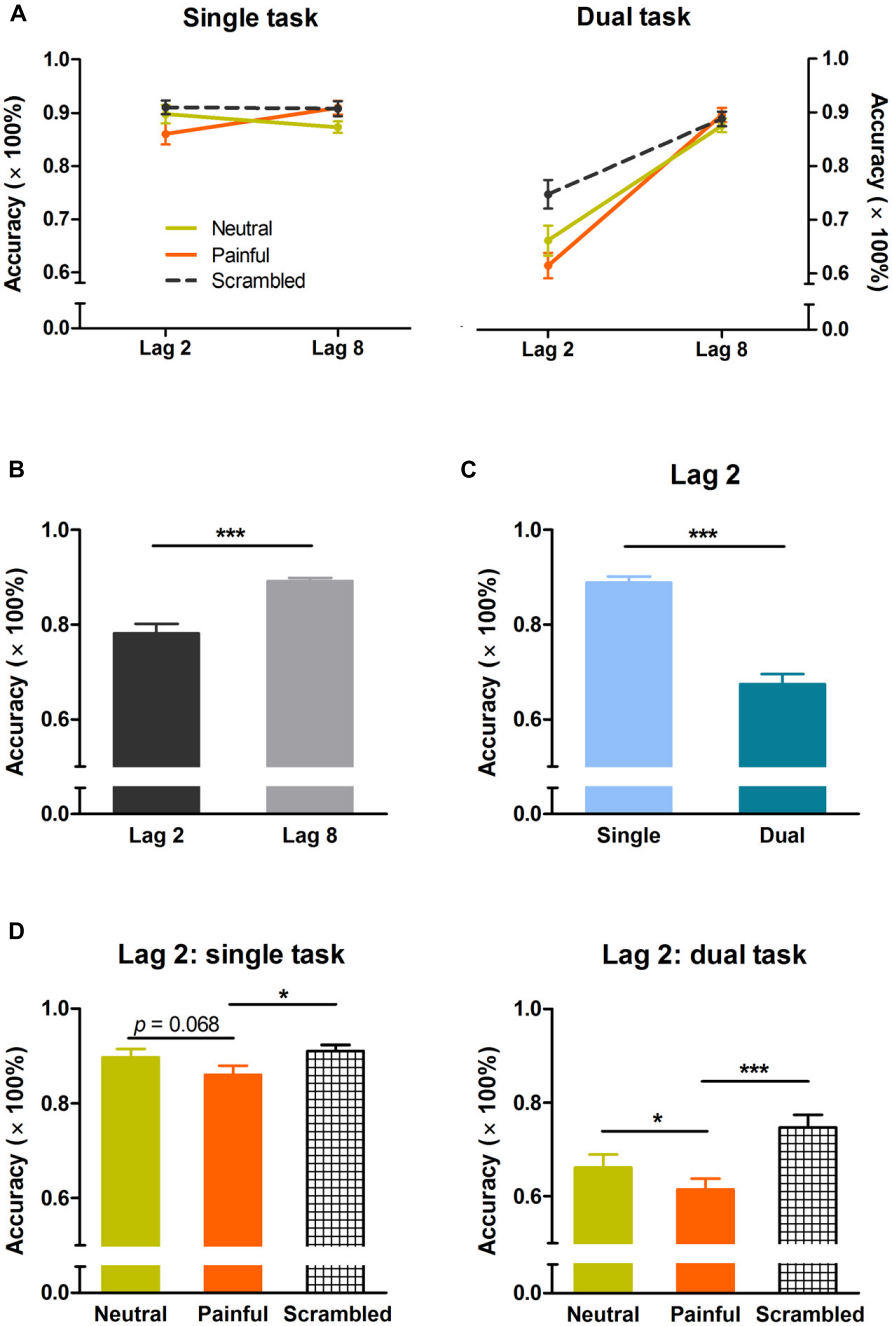
**Response accuracy in the single and dual tasks. (A)** Temporal distribution of target identification accuracy. **(B)** Overall effect of lag on response accuracy. **(C)** Effect of task relevance on response accuracy at lag 2. **(D)** Effects of distractor type on response accuracy at lag 2 in the single and dual tasks. Error bars indicate standard errors. *p < 0.05, ***p < 0.001.

Three-way ANOVA revealed significant interaction effects of task × lag (*F*_(1,50)_ = 82.29, *p* < 0.001, η^2^ = 0.62), lag × distractor type (*F*_(2,100)_ = 13.32, *p* < 0.001, η^2^ = 0.21), and task × lag × distractor type (*F*_(2,100)_ = 3.79, *p* < 0.05, η^2^ = 0.07). Significant main effects of task (*F*_(1,50)_ = 47.29, *p* < 0.001, η^2^ = 0.49), lag (*F*_(1,50)_ = 94.67, *p* < 0.001, η^2^ = 0.65), and distractor type (*F*_(2,100)_ = 11.58, *p* < 0.001, η^2^ = 0.19) were observed. **Figure 2A** shows the effect of distractor type on target identification accuracy according to target position (i.e., lag) for both tasks. Overall, mean accuracy was significantly lower at lag 2 than at lag 8 (78 ± 14 vs. 89 ± 5%, *p* < 0.001), indicating the presence of an AB effect (**Figure 2B**).

Two-way ANOVA (task × distractor type) of lag 2 data revealed significant task × distractor type interaction (*F*_(2,100)_ = 3.93, *p* < 0.05, η^2^ = 0.07), and main effects of task (*F*_(1,50)_ = 74.97, *p* < 0.001, η^2^ = 0.60) and distractor type (*F*_(2,100)_ = 16.21, *p* < 0.001, η^2^ = 0.25). The mean response accuracy was significantly higher in the single task than in the dual task, suggesting that the effect of interruption on target identification was more prominent for task-relevant than for task-irrelevant distractors (**Figure 2C**). *Post hoc* comparisons revealed significant differences between the single and dual tasks for all three distractor types (painful: 86 ± 10 vs. 61 ± 12%, *p* < 0.001; neutral: 90 ± 9 vs. 66 ± 14%, *p* < 0.001; scrambled: 91 ± 7 vs. 75 ± 14%, *p* < 0.001).

For both tasks, one-way ANOVA revealed significant main effects of distractor type at lag 2 (single task: *F*_(2,50)_ = 3.61, *p* < 0.05, η^2^ = 0.13; dual task: *F*_(2,50)_ = 13.76, *p* < 0.001, η^2^ = 0.36). *Post hoc* comparisons revealed a significant difference in target accuracy between painful and neutral conditions in the dual task (61 ± 12 vs. 66 ± 14%, *p* < 0.05), and a marginally significant difference in the single task (86 ± 10 vs. 90 ± 9%, *p* = 0.068; **Figure 2D**). Two-way ANOVA (task × distractor type) of lag 8 data showed a main effect of distractor type (*F*_(2,100)_ = 3.97, *p* < 0.05, η^2^ = 0.07), but no interaction and no effect of task.

### Correlations between Accuracy in Painful Distractor Trials and Questionnaire Scores

**FIGURE 3:**
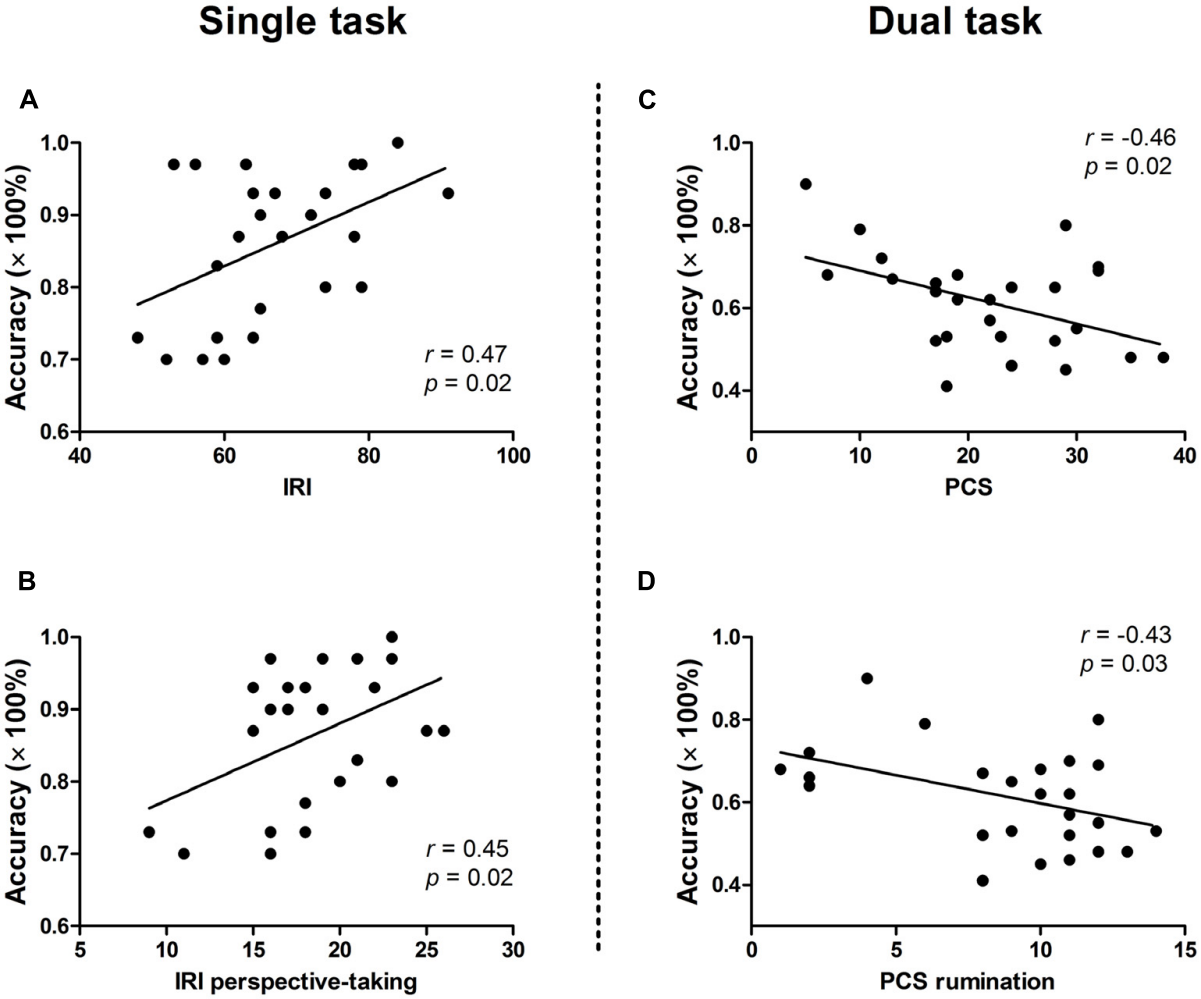
**Correlations between response accuracy in painful distractor trials (at lag 2) and personality traits**. Significant positive correlations were found between accuracy and IRI score **(A)** and IRI perspective-taking subscale score **(B)** in the single task. By contrast, significant negative correlations were observed between accuracy and PCS score **(C)** and PCS rumination subscale score **(D)** in the dual task. IRI, Interpersonal Reactivity Index; PCS, Pain Catastrophizing Scale.

Results of correlation analysis between response accuracy and individual variables of self-reported measures are presented in **Table 1**. In general, significant correlations were found only in painful trials, not in neutral trials. For the single task, target accuracy and IRI perspective-taking subscale score were positively correlated (*r* = 0.45, *p* < 0.05). The positive correlation between target accuracy and IRI total score (*r* = 0.47, *p* < 0.05) was due mainly to the perspective-taking subscale score. By contrast, for the dual task, significant negative correlations were observed between response accuracy and PCS total (*r* = −0.46, *p* < 0.05) and rumination subscale (*r* = −0.43, *p* < 0.05) scores. No other significant correlation was identified. Scatter plots of significant correlations between accuracy and questionnaire scores are presented in **Figure 3**.

**Table 1:** Mean scores of the questionnaires (Interpersonal Reactivity Index and Pain Catastrophizing Scale) and correlation coefficient values between the questionnaire and target accuracy (at lag 2).

	Single task			Dual task		
	Mean (SD)	Painful	Neutral	Mean (SD)	Painful	Neutral
**IRI total (0–112)**	67.04 (10.57)	0.47*	-0.01	69.92 (12.26)	0.16	0.22
*Perspective-taking*	18.12 (4.23)	0.45*	0.12	18.38 (4.32)	0.13	-0.18
*Fantasy*	16.27 (4.68)	0.29	0.10	16.50 (4.93)	0.20	0.22
*Empathic concern*	17.77 (3.71)	0.27	0.05	19.85 (3.94)	0.07	0.18
*Personal distress*	14.88 (4.68)	0.15	-0.28	15.19 (5.23)	0.02	0.32
**PCS total (0–52)**	16.77 (10.37)	0.10	0.01	21.85 (8.56)	-0.46*	-0.29
*Rumination*	8.65 (4.37)	0.18	0.10	8.77 (3.72)	-0.43*	-0.29
*Magnification*	3.27 (2.62)	0.02	-0.16	4.92 (2.61)	-0.25	-0.00
*Helplessness*	4.85 (4.58)	0.04	0.03	8.15 (4.72)	-0.36	-0.29

*p < 0.05; IRI, Interpersonal Reactivity Index; PCS, Pain Catastrophizing Scale.

## Discussion

The current study demonstrated that task-relevant painful faces had a significantly stronger AB effect (i.e., temporary visual processing impairment for targets appearing soon after faces) than did neutral faces, and task-irrelevant faces showed a near-significant trend of this effect. The attention-capturing effect was greater in the dual task than in the single task, consistent with our hypothesis. Importantly, we found that response accuracy for targets following painful faces was correlated positively with IRI perspective-taking subscale score in the single-task condition, and negatively correlated with PCS score in the dual-task condition.

Previous research has demonstrated that pain-related stimuli attract attention. Most evidence has come from studies of attentional bias, which have used paradigms such as the modified Stroop task (Muris et al., 1995; Roelofs et al., 2002), the dot-probe task (Keogh et al., 2001; Vervoort et al., 2011), and the spatial cueing task (Van Damme et al., 2004a, 2006a). In these studies, pain-related stimuli were always task-irrelevant cues, rather than task-relevant targets. Participants were not required to pay attention to the pain-related stimuli, and attention was captured by painful materials in a bottom–up fashion. In the present study, we used single-target and dual-target tasks to investigate the existence of a pain information-induced AB, and compared the bottom–up and top–down modulation of attention using task-irrelevant and task-relevant paradigms. Our results showed a significant pain information-induced AB effect (i.e., worse target performance in the painful condition than in the neutral condition) in the task-relevant condition, consistent with our hypothesis and with the findings of previous studies that emotional expressions or words can capture attention and interfere with an ongoing task (Mathewson et al., 2008; Srivastava and Srinivasan, 2010). As painful (negative emotional) expressions are more salient than neutral expressions, they obviously capture and hold attention more effectively, thereby causing more disruption of subsequent target identification.

Unexpectedly, we failed to find a significant pain information-induced AB effect in the task-irrelevant condition, although the results showed a near-significant trend of this effect. These results are not in accord with those of Most et al. (2005), who employed a single-task RSVP and found that negative images produced greater impairment of target processing than did neutral images. This inconsistency may be due to differences between studies in the intensity of negative emotional images. Most et al. (2005) used negative photographs of people and animals and included graphic images of violence, distress, and medical trauma. These bloody, violent scenes may produce a stronger emotional effect than did those depicting merely painful facial expressions in our study. Additionally, the high level of target accuracy (86%) at lag 2 in the painful condition indicates that the painful faces used in our study were less threatening than the images used by Most et al. (2005; who reported 71% accuracy). Furthermore, previous research has failed to demonstrate attentional bias toward pain-related words or images in healthy volunteers (Crombez et al., 2013). Most evidence supporting pain-related attentional bias has come from patients with chronic pain (Snider et al., 2000) or studies using conditioned visual signals of pain as cues (Crombez et al., 2013). Thus, the occurrence of attentional bias may depend on prior pain experience. In the present study, we used healthy subjects and found a marginally significant pain information-induced AB effect in the task-irrelevant painful condition, suggesting that the temporal attention paradigm is more sensitive than spatial attention paradigms for the measurement of pain-related attention-capturing effects.

As expected, we found that task-relevant painful faces interfered more with target identification than did task-irrelevant faces, suggesting that painful faces that were processed in a top–down manner engaged more attentional resources. The different effects of bottom–up and top–down attention to painful faces may be explained by the distinct temporal characteristics of these two systems. In the bottom–up pattern, attention is captured transiently by a pain distractor; in the top–down pattern, a painful face holds attention for a longer time and leaves limited resources available for target identification (Nakayama and Mackeben, 1989; Dux and Marois, 2009; Martens and Wyble, 2010; Pinto et al., 2013). Given that attention capturing was guided in the task-relevant condition compared with the task-irrelevant condition, target impairment in the dual task can be better explained by the holding, rather than capturing, of attention (Mathewson et al., 2008; Srivastava and Srinivasan, 2010). Additionally, when a subject is prepared to identify a target or has knowledge about the target while scanning an environment, relevant stimuli capture his/her attention more rapidly and accurately than if he/she was not prepared. This case may also reflect interplay between top–down and bottom–up processing. In real life, pain-related information is often not a currently pursued goal, but a signal of physical threat, for healthy individuals. It automatically attracts attention in a bottom–up fashion (Eccleston and Crombez, 1999). By contrast, physical symptoms have a persistent influence on the lives of patients with chronic pain, who must direct their attention toward physical pain or potential pain indicators in an attempt to control pain in a timely and efficient manner (Eccleston and Crombez, 1999, 2007; Van Damme et al., 2008). In this case, pain-related information is selectively attended to, and top–down processing may result in hypervigilance, i.e., increased arousal to environmental change.

In the present study, target performance was modulated by different individual traits in the two tasks. In the single-target task, response accuracy was correlated positively correlated with the IRI perspective-taking subscale; in the dual-target task, a negative correlation was found between response accuracy and catastrophic thinking about pain (particularly the PCS rumination subscale). The positive correlation between IRI perspective-taking subscale score and target performance is difficult to interpret, and may not be very meaningful. The painful faces used in this study did not likely evoke empathic responses. First, the photographs had a low degree of emotional strength. Second, the duration of stimulus presentation (100 ms) was much shorter than that typically used in empathy studies (≥200 ms; Fan and Han, 2008; Sessa et al., 2014). Finally, in the task-irrelevant condition, subjects were asked to ignore the distractor and the visual stimulus was processed only modestly. A possible explanation for this is that the perspective-taking subscale, which measures only the cognitive dimension of empathy, probably reflects general cognitive ability. That is, individuals with better ability to identify emotional faces had better task performance.

The negative correlation between target accuracy and catastrophizing thinking about pain was not surprising. In the dual task in which pain was task relevant, participants were required to focus on pain distractors, which may result in a greater degree of attention holding than in the task-irrelevant condition. Moreover, subjects who engage in more pain catastrophizing may have more difficulty disengaging their attention from pain-related stimuli. Our results fit well with the findings of Van Damme et al. (2002, 2004a) who demonstrated that catastrophizing thinking about pain predicted the retardation of disengagement from pain-related information using a cueing task (Van Damme et al., 2002) and a spatial cueing paradigm (Van Damme et al., 2004a). Previous studies have demonstrated that personality traits can modulate the AB by showing that greater extraversion and openness predicted smaller AB while greater neuroticism predicted larger AB (Maclean and Arnell, 2010). Our results also found the relationship between personality characteristics (i.e., catastrophizing thoughts) and AB in pain-face-as-target trials rather than neutral-face-as-target trials, suggesting that such a relation was specific to pain. Highly catastrophizing individuals may be inclined to extensive processing of pain, which may impede the disengagement of attentional resources from pain-related thoughts and feelings, thereby impairing the processing of other environmental stimuli (Vervoort et al., 2011).

## Conclusion

This study examined the attention-capturing effects of painful facial expressions using task-relevant and task-irrelevant AB paradigms. In the task-relevant condition, painful faces had a significant pain information-induced AB effect; task-irrelevant faces showed a near-significant trend of this effect. This difference was mediated by the distinct patterns of attentional processing (i.e., top–down vs. bottom–up), which may represent the different ways in which healthy individuals and patients with chronic pain process pain-relevant information. Moreover, in the dual-task condition, a higher level of pain catastrophizing predicted poorer task performance, suggesting that catastrophizing thoughts play a critical role in cognitive impairment in patients with chronic pain. These results extend previous findings by showing different effects of top–down and bottom–up processing of pain-related stimuli on cognitive function, and provide insight into maladaptive cognition in patients with chronic pain.

## Conflict of Interest Statement

The authors declare that the research was conducted in the absence of any commercial or financial relationships that could be construed as a potential conflict of interest.

## Abbreviations

AB: attentional blink
ANOVA: analysis of variance
IRI: Interpersonal Reactivity Index
PCS: Pain Catastrophizing Scale
RSVP: rapid serial visual presentation
SOA: stimulus onset asynchrony.
